# An Evaluation of Web- and Print-Based Methods to Attract People to a Physical Activity Intervention

**DOI:** 10.2196/resprot.4826

**Published:** 2016-05-27

**Authors:** Stephanie Alley, Cally Jennings, Ronald C Plotnikoff, Corneel Vandelanotte

**Affiliations:** ^1^ Physical Activity Research Group School of Human Health and Social Science Central Queensland University Rockhampton Australia; ^2^ Faculty of Physical Education and Recreation University of Alberta Edmonton, AB Canada; ^3^ Priority Research Centre for Physical Activity and Nutrition University of Newcastle Newcastle Australia

**Keywords:** physical activity, web-based intervention, Internet, research subject recruitment, Facebook

## Abstract

**Background:**

Cost-effective and efficient methods to attract people to Web-based health behavior interventions need to be identified. Traditional print methods including leaflets, posters, and newspaper advertisements remain popular despite the expanding range of Web-based advertising options that have the potential to reach larger numbers at lower cost.

**Objective:**

This study evaluated the effectiveness of multiple Web-based and print-based methods to attract people to a Web-based physical activity intervention.

**Methods:**

A range of print-based (newspaper advertisements, newspaper articles, letterboxing, leaflets, and posters) and Web-based (Facebook advertisements, Google AdWords, and community calendars) methods were applied to attract participants to a Web-based physical activity intervention in Australia. The time investment, cost, number of first time website visits, the number of completed sign-up questionnaires, and the demographics of participants were recorded for each advertising method.

**Results:**

A total of 278 people signed up to participate in the physical activity program. Of the print-based methods, newspaper advertisements totaled AUD $145, letterboxing AUD $135, leaflets AUD $66, posters AUD $52, and newspaper article AUD $3 per sign-up. Of the Web-based methods, Google AdWords totaled AUD $495, non-targeted Facebook advertisements AUD $68, targeted Facebook advertisements AUD $42, and community calendars AUD $12 per sign-up. Although the newspaper article and community calendars cost the least per sign-up, they resulted in only 17 and 6 sign-ups respectively. The targeted Facebook advertisements were the next most cost-effective method and reached a large number of sign-ups (n=184). The newspaper article and the targeted Facebook advertisements required the lowest time investment per sign-up (5 and 7 minutes respectively). People reached through the targeted Facebook advertisements were on average older (60 years vs 50 years, *P*<.001) and had a higher body mass index (32 vs 30, *P*<.05) than people reached through the other methods.

**Conclusions:**

Overall, our results demonstrate that targeted Facebook advertising is the most cost-effective and efficient method at attracting moderate numbers to physical activity interventions in comparison to the other methods tested. Newspaper advertisements, letterboxing, and Google AdWords were not effective. The community calendars and newspaper articles may be effective for small community interventions.

**ClinicalTrial:**

Australian New Zealand Clinical Trials Registry: ACTRN12614000339651; https://www.anzctr.org.au/Trial/Registration/TrialReview.aspx?id=363570&isReview=true (Archived by WebCite at http://www.webcitation.org/6hMnFTvBt)

## Introduction

Recent reviews and meta-analyses have confirmed the short-term effectiveness of Web-based physical activity interventions [1-3]. However the public health impact of these interventions is dependent on how many people they reach when disseminated. It is more difficult and complex to successfully attract people to participate in Web-based health programs than may commonly be perceived. The Internet is a very competitive environment with hundreds of thousands of websites all vying to attract the attention of a potentially vast audience. To date, a range of methods have been used to attract participants to Web-based health interventions including newspaper advertisements [4], leaflets [5], email [6], and social media [7]. However, limited research has measured the cost-effectiveness of these methods [8,9].

Traditional methods including leaflets, posters, and newspaper advertisements are still commonly used by researchers and health professionals to attract people to Web-based heath interventions. This is despite the growing use of Web-based methods, which have the potential to reach larger numbers at lower cost [8]. Email has been successfully used to attract specific groups such as workplace employees to Web-based health interventions when email lists are available [9]. Email is nevertheless inappropriate for a population-wide dissemination as it is becoming increasingly hard to engage people with emails that are not targeted and personally relevant, or not from a trusted source [10]. Internet banners, links on websites, social media, and search engines are popular and effective Web-based marketing methods in the commercial sector [11].

Recent studies have successfully used social media to attract adolescents and adults to nutrition, smoking, and other health interventions [12-14]. Gilligan et al [7] found Facebook advertisements to be 10 times more cost-effective at recruiting mothers of adolescents to complete a survey than traditional media. Furthermore, Morgan et al [15] found Google AdWords to be a cost-effective method for attracting adults to a depression intervention across 6 western countries. Their results also revealed that posts in forums and community notice boards were not effective. A better understanding of how the Internet can be used to attract people to Web-based heath interventions will help researchers and public health workers attract large numbers to Web-based health interventions. Therefore, evaluating the cost-effectiveness of Web- and print-based methods to promote Web-based health interventions requires investigation.

Participants of Web-based physical activity interventions reached through traditional print-based methods have typically been Caucasian, female, and educated [16,17]. Internet use in Australia is currently widespread (83%); however, Internet use decreases with lower education and income [18]. It is unknown whether Web-based advertising is more effective at reaching a representative sample than print-based advertising. Facebook advertising provides the advantage of targeted advertising to assist in reaching desired sample characteristics [12,19]. Therefore, further research is required to determine the characteristics of people reached through different print- and Web-based methods for promoting Web-based health interventions.

This study aims to determine the effectiveness (in terms of cost, time investment, and numbers reached) of multiple Web- and print-based methods to attract Australian adults to a Web-based physical activity intervention. It is hypothesized that the Google AdWords and Facebook advertising will reach a larger number of people at a lower cost and time investment per sign-up than the community calendar and the traditional print-based methods.

## Methods

The current study recorded and analyzed methods to attract people to a Web-based physical activity intervention trial in Australia. Data collection began in March 2014. The intervention itself was part of a randomized controlled trial (RCT) comparing the effectiveness (in terms of engagement, retention, and physical activity changes) of computer-tailored advice only and computer-tailored advice with a brief coaching session in an 8-week intervention. A detailed description of the intervention, measures, and study protocol has been published elsewhere [20]. Participants were excluded from the RCT if they were non-English speaking, pregnant, under 18 years of age, currently meeting the Australian physical activity guidelines (assessed by a single item, “do you currently participate in less than 30 minutes of physical activity on average each day?”), or at risk of injury or ill health from increasing their physical activity (assessed by the Physical Activity Readiness Questionnaire [21]). These criteria are stricter than typical population- or community-based physical activity interventions, so all interested participants’ data were included in the current study, regardless of their eligibility to participate in the following RCT. These numbers are more likely to reflect the effectiveness of strategies to attract people to real-world Web-based interventions that aim to sign up as many people as possible. The research has been approved by the Central Queensland University Human Ethics Committee (H13/04-044). Informed consent to participate in the study was obtained from all participants.

### Stage 1 Sign-Up

The advertising methods were implemented in the cities of Rockhampton and Mackay, Queensland, Australia. All print-based advertisements including a newspaper advertisement, posters, and leaflets displayed the intervention logo, the CQUniversity logo, the intervention URL and a quick response (QR) code. The QR code directs people to the intervention website when they scan it with a smartphone. Readers were asked “Do you want to get healthy and fit and go in the draw to win some fabulous prizes? You are invited to participate in an online research study where you will gain free access to the ‘My Activity Coach’ program developed by CQUniversity. It will provide you with personalized advice to help you become more active.” The 10x3 cm newspaper advertisements were printed in a local newspaper (the Morning Bulletin) that has a circulation of 20,000 in the Rockhampton region. The newspaper advertisements contained the same information as the posters and leaflets but in a more concise format, due to space restrictions. A graphic designer assisted with the design of the print advertisements.

**Figure 1:**
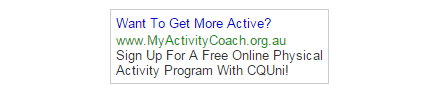
Google advertisement.

The Web-based methods included paid advertisements targeted to Mackay residents on the social media website Facebook and on the Google search engine (Google AdWords). Advertisements were also displayed on Mackay’s community websites, including the local newspaper’s website (the Daily Mercury) and My Community Connect Mackay at no cost. These advertisements listed the program in the websites’ event calendars. Participants could click on the calendar entry to find out more information including a link to the intervention website. The Google advertisements appeared when users searched for terms related to the intervention including fitness, healthy, physical activity, weight loss, and exercise. The Google search engine displays the advertisements that generate the largest number of clicks for each keyword searched. Therefore, each advertisement is competing against similar advertisements linked to the same keyword(s). The advertisement displayed one sentence about the program (see Figure 1) and took people who clicked on the advertisement directly to the intervention website. Multiple advertisements were trialed with different wording. The frequency of the advertisements that generated the largest number of clicks was increased. A daily maximum spend of AUD $10 was applied.

**Figure 2:**
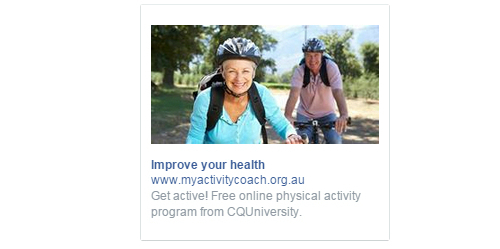
Facebook advertisement.

Facebook advertising allows clients to create an advertisement with text and a picture that can be targeted to Facebook user’s demographics and interests. The advertisements can be displayed as part of the user’s newsfeed, or on the right side of their newsfeed. If the Facebook users click on the feed advertisement, they will be taken to the organization’s Facebook page, and if the users click on the side advertisement they will be taken directly to the organization’s website. Multiple advertisements can be created within a campaign, with the option to target each advertisement to specific demographics such as age, gender, and location, and/or to people who have shown interest in certain Facebook pages. Only side advertisements were used in the current study to promote direct traffic to the intervention website. Only Facebook users who were over 18 and reside in Mackay were targeted. No additional targeting was implemented to avoid biasing the sample of people reached. Multiple advertisements were trialed with different wording and pictures. Facebook increases the frequency of advertisements that generate the largest number of clicks from the target audience. A limit of AUD $20 per day was applied. The Facebook advertisements displayed a picture and a sentence inviting people to participate in the physical activity intervention (see Figure 2). Both the Facebook advertisements and Google AdWords were checked by an online marketing specialist.

### Stage 2 Sign-up

#### Stage 2a

At the end of May 2014 after 2 months of promoting the intervention, only 22 people had completed the sign-up survey. To increase the number of sign-ups, the advertising methods were extended to include several other Australian towns: Bundaberg, Townsville, and Brisbane in Queensland; Melbourne (Victoria), Perth (Western Australia), and Sydney (New South Wales). The newspaper advertisements and Google AdWords that had a very high cost per sign-up were discontinued. A new method of delivering leaflets to people’s homes was also implemented.

#### Stage 2b

**Figure 3:**
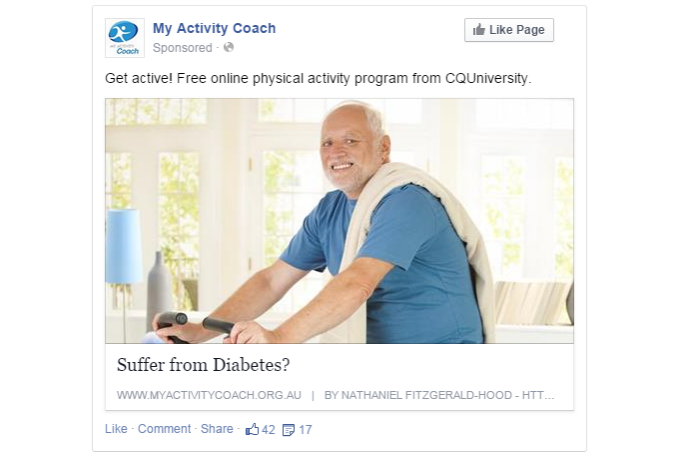
Feed advertisement targeted to males over 45 years with diabetes.

By August 15, 2014, only 61 people had completed the sign-up survey. Delivering leaflets to people’s homes had a very high cost and time investment, so this was discontinued. Furthermore, no additional posters and leaflets were distributed through health care centers. The event listings in the online calendars, however, were continued as they were free and quick to implement. A news article about the program was printed in the Morning Bulletin on September 1, 2014; this was free of cost and took only 1.5 hours to arrange with the newspaper staff. The costly untargeted Facebook advertising was also discontinued. Instead, new highly targeted Facebook advertising was implemented across Australia. From this point forward, this was the main recruitment method (see Table 1). The advertisements were targeted to gender, whereby the advertisements showed a photo of an active person of the same gender. Furthermore, in order to reach individuals most likely to be interested and with the most to gain from the intervention, individuals who had diabetes, depression, cancer, and heart disease were targeted. To do this, people who were members of Facebook pages and support groups on diabetes, depression, cancer, and heart disease were targeted. These advertisements included a statement relating to the condition the user was connected with. Finally some Facebook advertisements were shown only to individuals over 45 years of age. These advertisements displayed a picture of an active older person. Both feed advertisements that are displayed on the user’s newsfeed alongside posts from their Facebook friends and groups, and side advertisements displayed on the right border of the Facebook webpage were used. Different combinations of gender, age, disease, and advertisement location were created resulting in 24 different advertisements (eg, feed advertisement targeted to males over 45 years with diabetes; Figure 3). Initially, a daily maximum spend of AUD $5 for each advertisement was selected, but this was regularly updated by increasing the maximum daily spend for the advertisements that had the lowest cost-per click to the intervention website.

**Table 1:** Timeline of strategies used to attract people to the intervention website.

Strategies	2014										2015			
	Mar	Apr	May	June	July	Aug	Sep	Oct	Nov	Dec	Jan	Feb	Mar
		Rockhampton and Mackay in QLD				Rockhampton, Mackay, Bundaberg, Townsville and Brisbane in QLD; Melbourne (VIC), Perth (WA), and Sydney (NSW)								
Facebook	√	√	√	√	√							
Google	√	√	√									
Newspaper advertisement	√	√	√									
Calendar^a^	√	√	√	√	√	√	√	√	√	√	√	√	√
Leaflet	√	√	√	√	√	√	√	√	√	√	√	√	√
Poster	√	√	√	√	√	√	√	√	√	√	√	√	√
Letter^b^				√	√							
Newspaper article							√					
Targeted Facebook						√	√	√	√	√	√	√	√

^a^Calendar=online community calendar.

^b^Letter=letterbox drop.

### Measures

#### Participant Numbers

The number of times each advertisement was displayed (impressions) were recorded for the Facebook and Google AdWords advertisements. For the newspaper advertisements, the circulation (estimated number of readers) was used as the measure of impressions. The number of first time website visits from each of the Web-based methods was recorded through Google analytics and the number of website visits for each of the 24 targeted Facebook advertisements was recorded through Facebook. After visiting the homepage, individuals were encouraged to complete a screening survey that asked participants, “how did you hear about this program?” Options included “Google search,” “Facebook,” “community calendar,” “newspaper article,” “newspaper advertisement,” “leaflet,” “letterbox drop,” “poster,” or “other.” This was used as the measure of sign-ups. Generating sign-ups is the main goal of the advertisements and is likely to reflect the number of participants reached though real-world interventions that do not have strict eligibility criteria. Participants who selected “other” were excluded from the current analysis.

#### Cost

The total cost of each of the methods used to attract people to the intervention was calculated. This included the cost of a research assistant to implement each method based on the Central Queensland University rate of AUD $35/hr. The money spent on each of the 24 targeted Facebook advertisements was also recorded through Facebook. The time spent implementing each method was also calculated. This included the time spent planning, executing, and monitoring each advertisement.

#### Demographic Characteristics

Participant demographics were assessed only for those people who signed up, were eligible, and who completed the baseline survey (140 out of 278 that signed up). The questionnaire collected participant demographics including gender, marital status, language, income, education, employment, age, body mass index (BMI), and physical activity. Total minutes of physical activity during the previous week was assessed by the validated Active Australia Questionnaire [22].

### Data Analysis

#### Data Screening

All analyses were conducted using SPSS version 20. Significance level was set at *P*<.05. All continuous variables were screened for outliers and normality using Fisher’s skewness coefficient. Age, BMI, and total physical activity per week were found to have a significant negative, positive, and positive skewed distribution respectively. A reflect and square root logarithm and square root transformation successfully transformed these variables into normal distributions respectively.

#### Participant Numbers

The number of impressions, first time visits to the website, and sign-ups for each advertising method were calculated.

#### Cost

Money spent on the recruitment method plus the hours spent implementing the recruitment method times the hourly cost of employing a research assistant (AUD $35) was calculated and divided first by the number of website visits and second, the number of sign-ups from each recruitment method. Cost per sign-up was the main outcome measure. The time invested in each advertising method per sign-up was calculated as a measure of efficiency.

#### Demographic Characteristics

The demographic characteristics of participants reached through the Web-based and print-based recruitment methods were calculated and compared using chi-square analysis for the categorical characteristics (gender, language, marital status, employment, education, and income) and analysis of variance for the continuous characteristics (age, BMI, and physical activity).

## Results

### Participant Numbers

**Figure 4:**
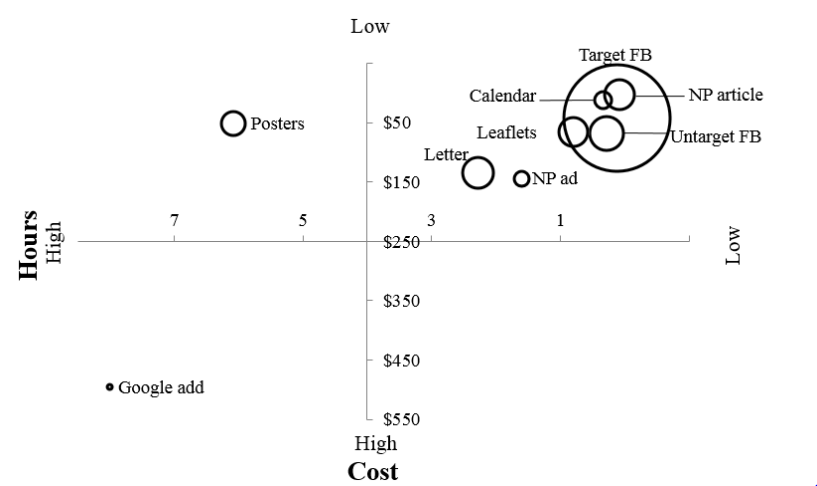
Hours per sign-up, cost per sign-up, and number of sign-ups for each advertising method.

The strategies implemented in Stage 1 led to 59 sign-ups, and the letterboxing implemented in Stage 2a led to 18 sign-ups. During Stage 2b, the targeted Facebook recruitment led to 184 sign-ups and the newspaper article led to an additional 17 sign-ups (see Table 2 and Figure 4).

### Cost

Two newspaper advertisements were printed, which totaled AUD $446. A total of 8 hours was spent organizing the newspaper advertisements costing $280 in research assistant time. In total, 1000 leaflets and 150 posters were displayed in 20 health care centers throughout Rockhampton, and 21 health care centers in Brisbane. The leaflets and posters cost AUD $570.35 and AUD $154 for printing respectively. A total of 24 hours were spent distributing the leaflets and posters, costing AUD $840. Another 3500 and 1500 leaflets were delivered to homes in Rockhampton and Melbourne respectively. The printing for these leaflets cost AUD $990. A total of 41 hours were spent delivering the leaflets, costing $1435.

The calendar entry in the Daily Mercury’s website and Mackay’s My Community Connect was free of costs. However, the research assistant spent 2 hours organizing this, costing $70. A total of AUD $215 was spent on Google AdWords. The research assistant spent 8 hours developing, implementing, and evaluating the Google advertisements, costing $280. Untargeted side Facebook advertisements totaled $1228. The research assistant spent 6 hours developing, implementing, and monitoring these Facebook advertisements, costing AUD $210. The targeted Facebook advertisements totaled AUD $7021. The research assistant spent 20 hours developing, implementing, and monitoring these Facebook advertisements, contributing AUD $700 to the total cost (see Figure 4).

**Table 2:** Time investment, costs, impressions, first time visits, and sign-ups of each advertising method.

	Advertising method	Time, hrs	Cost, $	Impressions, n	Visits, n	Sign- ups, n	Time per sign-up, mins	Cost per impression, $	Cost per sign-up, $
**Web-based**									
	Community calendar	2	70	‒	20	6	20	‒	12
	Untargeted Facebook	6	1438	119,806	877	21	17	0.01	68
	Targeted Facebook	20	7721	547,507	5372	184	7	0.01	42
	Google AdWords	8	495	18,773	34	1	480	0.03	495
**Print**									
	Posters	12	574	–	–	11	365	–	52
	Health care leaflets	12	990	–	–	15	48	–	66
	Letterbox drop	41	2425	6000	–	18	137	0.4	135
	Newspaper ad	8	726	40,000	–	5	96	0.02	145
	Newspaper article	1.5	53	20,000	–	17	5	0	3
Total		110.5	14,492	752,086	6303	278	1175	0.08	113.11

### Demographic Characteristics

A total of 140 participants out of 278 (50.4%) completed the baseline questionnaire. The remaining 49.6% did not meet the inclusion criteria for the RCT (inactive Australian over 18 years with no health conditions that may affect their ability to safely become more active) or failed to complete the baseline questionnaire. This comparison was conducted instead of comparing all print methods to Web-based methods as there was a large variance in effectiveness of the Web-based methods and targeted Facebook advertisements were the most effective method at reaching large numbers at low cost. People reached through the targeted Facebook advertisements were on average older and had a higher BMI than people reached through the other methods. They were also more likely to be divorced or widowed and less likely to have never married, which is likely to be due to the older age group (see Table 3).

**Table 3:** Descriptive summary of participant characteristics reached using targeted Facebook advertisements and all other methods.

Participant characteristics		Targeted Facebook (n=74)	All other methods (n=66)	Comparison
Gender, n (%)				χ^2^=1.24, *P*=.27
	Males	19 (26)	12 (18)	
	Females	55 (74)	55 (82)	
First language, n (%)				χ^2^=0.26, *P*=.61
	English	71 (97)	66 (98)	
	Other	2 (3)	1 (2)	
Marital status, n (%)				χ^2^=6.17, *P*=.01
	Never married	0 (0)	5 (7)	
	Married	50 (69)	46 (69)	
	Divorced or widowed	23 (31)	16 (24)	
Employment, n (%)				χ^2^=3.23, *P*=.19
	Full time	24 (33)	29 (43)	
	Part time/casual	13 (18)	15 (22)	
	Unemployed	36 (49)	23 (35)	
Education, n (%)				χ^2^=1.55, *P*=.46
	Less than secondary	1 (1)	1 (2)	
	Secondary	13 (18)	7 (10)	
	TAFE or university	59 (81)	59 (88)	
Income, AUD, n (%)				χ^2^=1.4, *P*=.50
	Over $78,000	24 (49)	28 (50)	
	$31,200-77,999	13 (26)	19 (34)	
	Under $31,199	12 (25)	9 (16)	
Age in years, mean (SD)		59.72 (.57)	50.21 (1.24)	F_1,141_=57.20, *P*<.001
BMI (kg/m^2^), mean (SD)		32.0 (.83)	30.36 (1.04)	F_1,139_=5.081, *P*=.07
Total physical activity (minutes/wk), mean (SD)		161.67 (22.07)	162.84 (24.19)	F_1,141_=0.05, *P*=.83

### Targeted Facebook Advertising

**Figure 5:**
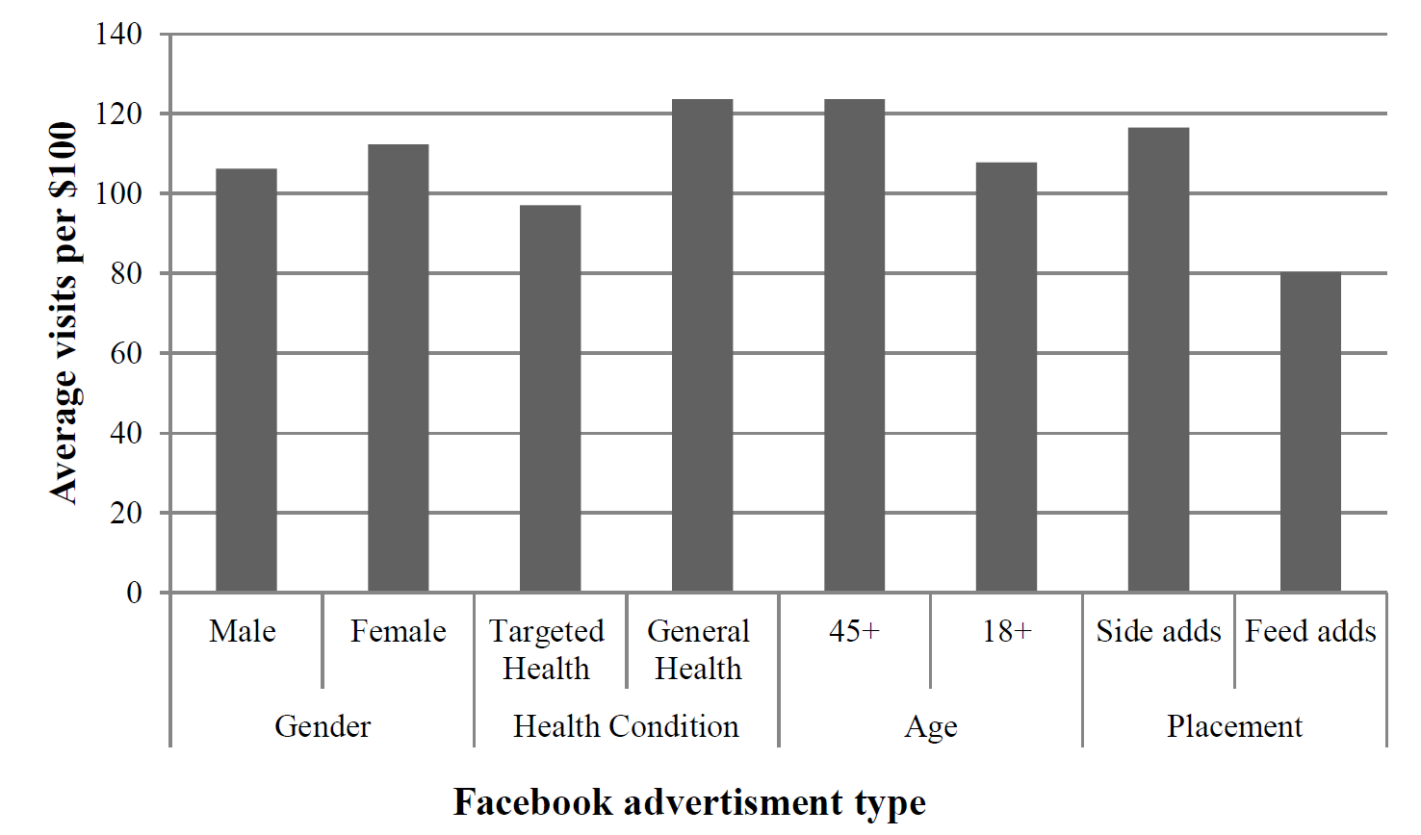
Average number of visits per $100 spent on each Facebook advertisement target.

The three most cost-effective Facebook advertisements at bringing people to the intervention website were (1) a feed advertisement targeting males over 45 years with diabetes, (2) a side advertisement targeting females over 45 years, and (3) a side advertisement targeting females over 18 years. The female targeted advertisements were more cost-effective per website visit than the male targeted advertisements except for the diabetes, depression, and heart-targeted advertisements. The advertisements targeted to adults over 45 years were more cost-effective per website visit than the advertisements shown to all ages. The advertisements targeted to general health were more cost-effective as a side advertisement, while the targeted health advertisements were more cost-effective as a feed advertisement. Overall, the side advertisements were more cost-effective per website visit than the feed advertisements, except for the diabetes-targeted feed advertisement (see Table 4). The advertisements targeting females were more cost-effective than the advertisements targeting males. The advertisements targeting general health were more cost-effective than the advertisements targeting specific diseases, and the advertisements targeting adults over 45 years were more cost-effective than the advertisements targeting adults over 18 years (see Figure 5).

**Table 4:** Cost, impressions, website visits, and sign-ups for each Facebook advertisement ordered by cost per visit.

Facebook advertisement	Real cost, $	Outcomes			Cost per outcome	
		Impressions	Visits	Sign-ups	Cost per impression, $	Cost per visit, $
Feed^a^, diabetes, male	85.49	3805	127	2	0.02	0.67
Side, health^b^, female	2500.92	259,992	3237	53	0.01	0.77
Side, health, female, all ages	646.91	175,051	762	0	0.00	0.85
Side, health, male, all ages	123.72	65,069	144	0	0.00	0.86
Side, health, male	566.64	113,949	648	9	0.00	0.87
Side, cancer, female	144.44	19,456	165	1	0.01	0.88
Feed, diabetes, female	315.09	11,828	354	9	0.03	0.89
Side, diabetes, male	241.46	3111	268	2	0.08	0.90
Side, depression, male	38.06	3901	39	0	0.01	0.98
Side, diabetes, female	564.05	8051	547	2	0.07	1.03
Side, heart, male	215.47	6141	210	1	0.04	1.03
Side, heart, female	590.06	13148	569	7	0.04	1.04
Side, depression, female	87.72	7310	84	0	0.01	1.04
Side, cancer, male	29.49	5043	24	0	0.01	1.23
Feed, cancer, female	103.17	5123	72	0	0.02	1.43
Feed, heart, female	111.42	4663	65	0	0.02	1.71
Feed, depression, female	116.73	5054	67	0	0.02	1.74
Feed, health, female	93.03	6400	48	15	0.01	1.94
Feed, health, female, all ages	80.34	9584	41	0	0.01	1.96
Feed, health, male	32.67	3709	16	1	0.01	2.04
Feed, heart, male	14.18	1019	6	1	0.01	2.36
Feed, health, male, all ages	45.36	6,037	19	0	0.01	2.39
Feed, cancer, male	22.61	1382	8	0	0.02	2.83
Feed, depression, male	8.98	735	3	0	0.01	2.99
Can’t remember	–	–	–	37	–	–
Total	6778.01	739,561	7523	140	0.01	0.90

^a^Feed advertisements are displayed on the user’s newsfeed alongside posts from their Facebook friends and groups, and side advertisements are displayed on the right border of the Facebook webpage.

^b^Health refers to advertisements that were not targeted to a physical activity‒related chronic disease.

## Discussion

### Principal Findings

The effectiveness of the Web-based strategies hypothesized to be the most effective (Facebook and Google AdWords) was highly varied. The results demonstrate the low cost-effectiveness of the Google AdWords (AUD $495), while the targeted Facebook advertisements were the most cost-effective (AUD $42 per sign-up) method at reaching a large number of sign-ups (n=184). This is line with previous research demonstrating Facebook to be a cost-effective method for recruiting adolescent and young adults to nutrition and smoking interventions in Australia and North America [12-14]. The effectiveness of Facebook advertisements per sign-up may be due to the low cost per impression and the ability to tailor advertisements to individuals who are more likely to be interested in a Web-based physical activity intervention (eg, females over 45 years). The Facebook advertisements were not as effective when the targeting was not used, confirming that the effectiveness of the Facebook advertisements is partially due to tailoring. Health professionals implementing Facebook advertising in future interventions should target the advertisements to reduce costs; however, they need to be aware that this can bias the sample reached. Researchers and public health professionals must balance cost-effectiveness with how representative the sample reached through Facebook advertising will be. On the other hand, targeted Facebook advertisements can be used to reach underrepresented demographics (eg, people with a mental health condition) or those most in need of a physical activity intervention (eg, males, people over 45 years), but this may result in a higher cost per click. Although the Facebook advertising was the most cost-effective method at reaching moderate numbers, AUD $42 is still too costly for population-wide dissemination of interventions. Facebook advertising may need to be used in conjunction with mass media and viral marketing strategies to be cost-effective for large-scale disseminations [23]. Overall, the findings from the current study provide evidence to support the use of targeted Facebook advertising to attract people to a Web-based physical activity intervention.

Facebook advertisements displayed on the right side panel that target females over 45 years and general health were generally more cost-effective at attracting people to the physical activity intervention website. It is not surprising that the advertisements targeted to older females are the most cost-effective as this is the demographic that is typically more interested in physical activity interventions [16,17]. In many physical activity interventions, women outnumber men 60% to 40% [1]. Furthermore, it is not surprising that the smaller side advertisements were more cost-effective than the feed advertisements. The feed advertisements resulted in more clicks per dollar spent. However, the feed advertisements take users to another Facebook page (about the physical activity intervention) and then users need to click again to go to the intervention website itself. It is therefore likely that many people were lost at this additional step. It is surprising, however, that the advertisements targeted to people who had liked Facebook pages about diabetes, heart disease, depression, or cancer were not as effective as the advertisements targeted to general health. This could be due to the people who had liked these pages not having the disease themselves. Alternatively it could be due to a small number of users fitting the target criteria, resulting in a high number of impressions made to the same people (ie, repeated displays of the same advertisement to the same uninterested users).

Participants were asked in the sign-up survey which Facebook advertisement allowed them to find out about the program. Due to 37 out of 140 participants’ “not remembering” (26.4%), we did not include the analysis of cost per sign-up for each Facebook advertisement. The result of this analysis suggests that the feed advertisements targeted to specific diseases were the most cost-effective at recruiting participants. This means that more people clicked on the side advertisements per dollar spent, but many decided they were not interested after visiting the intervention website and did not sign up. On the other hand, fewer people clicked on the feed advertisements per dollar spent, but a higher percentage of these people signed up to the intervention making it the more cost-effective for achieving sign-ups. Although we cannot draw any conclusions from this data, it highlights the importance of future interventions to monitor website visits as well as sign-ups to evaluate the performance of each Facebook advertisement. Facebook now allows customers to do this by choosing a “conversion” behavior (eg, clicking on the sign-up button) to automatically track how many people from each advertisement begin the sign-up questionnaire on the website.

The high cost and low effectiveness of Google AdWords was not expected as Google AdWords is an effective marketing strategy in the commercial sector, and the advertisements were shown to people who were searching for information related to the intervention. This means that the advertisements were viewed by people more likely to be interested in physical activity and health, compared to Facebook or print-based advertisements, which are shown to people who are not searching for information on physical activity and health. Further, these findings are inconsistent with Morgan et al [15] who found Google AdWords to be a cost-effective method for attracting adults to a depression intervention across 6 western countries ($12 per participant). The high cost of the Google AdWords for the current physical activity program could be due to a high level of competition for the AdWords used including exercise and weight loss. Google increases the frequency of the advertisements that generate the most clicks. Our Google advertisements were therefore competing against commercial weight loss and exercise-related companies in the multibillion dollar fitness industry, which are more likely to gain clicks and therefore be shown more frequently.

The newspaper article and calendar cost the least per sign-up (AUD $3 and AUD $12 respectively); however, they reached only a small number of sign-ups (n=6 and 17 respectively). This is in line with the findings from Morgan et al [15], that community forums and notice boards resulted in few participants. Furthermore, it may not be possible for state- or national-wide interventions to use community forums and calendars as many accept only local events. The only forums that accepted the advertisement for the current project were in Mackay, as Mackay has a Central Queensland University campus through which the program was run. The newspaper article was easy to arrange in Rockhampton where the main campus of the university is located; however, newspapers in other towns were not interested. Although Web-based community forums and newspaper articles did not reach many people, small community interventions may benefit from these methods as they can reach a few additional people for a minimal time and monetary investment.

It is not surprising that the newspaper article and Facebook advertisements required the least amount of time investment per sign-up. The newspaper article was organized by newspaper staff at their expense, so the only time investment was contacting them about the program and answering some interview questions over the phone. The tailored Facebook advertisements can be set up online without any face-to-face meetings or need to manually distribute. Only a small time investment was required to choose appropriate figures and wording for the advertisements and to monitor and adjust them when they were running. The other advertisements needed to be distributed to health clinics (posters and leaflets), delivered to people homes (leaflets), and arranged with face-to-face meetings (newspaper advertisement). This further supports the use of targeted Facebook advertising to attract large numbers to Web-based physical interventions with a wide reach.

There are currently 12 million Facebook users in Australia [24], and the demographics of Facebook users has broadened in recent years [25]. Due to the wide reach of Facebook, Facebook advertising has reached a representative sample of the target population in many Australian studies [7,26]. It was therefore surprising that people reached through the targeted Facebook advertisements in our study were significantly older than those reached through the other methods. This may have been influenced by the Facebook advertisements that were targeted to people over 45 years. These advertisements were displayed more frequently as they resulted in a lower cost per click. The high prevalence of chronic conditions related to physical inactivity in older adults (eg, osteoarthritis, cardiovascular disease, diabetes, and falls), and the aging population means there is a specific need for physical activity interventions that can reach older adults [27]. The finding that participants reached through targeted Facebook advertising had a significantly higher BMI than participants reached through the other methods may be due to the advertisements targeting individuals with cardiovascular disease and diabetes. It is encouraging that the findings of this study demonstrate the capability of Web-based methods at reaching people most in need of a physical activity intervention.

### Limitations

This study presents data to help researchers and public health professionals understand the cost, effectiveness, and issues surrounding different methods to attract people to a Web-based health intervention. However, the findings have limitations, including that the demographic data were collected only for participants eligible to participate in the RCT and who completed the baseline assessment. Thus, it did not include people who signed up but were not eligible. Further, to ensure enough people participated in the RCT, the amount of funds allocated to the advertisements was continuously evaluated and directed to the more successful advertisements over the recruitment period (hence Stages 1, 2a, and 2b during the recruitment phase). Therefore, the most successful advertisement method (eg, Facebook ads) was used for longer than the non-successful advertisements (eg, Google AdWords and newspaper ads). The differences in time of year and total amount spent on different advertisement methods may have affected their success.

### Conclusion

Our findings reveal that targeted Facebook advertising is the most cost-effective method to attract moderate numbers to a Web-based health intervention while Google AdWords, despite being a popular marketing method in the commercial sector, was the least cost-effective method. However, the cost of Facebook advertisements are unsustainable for large population-based interventions that seek widespread implementation. Such interventions may need to use mass media in addition to Facebook advertising to reach larger numbers at a lower cost. Community calendars and newspaper articles were the cheapest methods; however, they reached a limited number of sign-ups. These interventions may therefore be beneficial for local community-based interventions. In summary, our findings suggest that Facebook advertising is the most cost-effective method at attracting moderate numbers to physical activity interventions in comparison to the other methods tested. However, it is still too costly for population-based interventions. Further research is needed to determine alternative recruitment procedures more effective at reaching large numbers of participants at low cost.

## Abbreviations

BMI: body mass index
QR: quick response
RCT: randomized controlled trial
